# Impact of the Mediterranean Diet on Glycemic Control, Body Mass Index, Lipid Profile, and Blood Pressure in Type 2 Diabetes: A Meta-Analysis of Randomized Controlled Trials

**DOI:** 10.3390/nu17243908

**Published:** 2025-12-13

**Authors:** Ming-Ju Wu, Cheng-Hsien Hung, Su-Boon Yong, Gregory S. Ching, Heng-Ju Hsu

**Affiliations:** 1 School of Chinese Medicine, China Medical University, Taichung 406040, Taiwan; 096014raymond@gmail.com (M.-J.W.); harry95218@gmail.com (H.-J.H.); 2 Department of Medicine, College of Medicine, China Medical University, Taichung 406040, Taiwan; yongsuboon@gmail.com; 3 Department of Pharmacy, Chang Bing Show Chwan Memorial Hospital, Changhua 505029, Taiwan; 4 Department of Allergy and Immunology, China Medical University Children’s Hospital, Taichung 404333, Taiwan; 5 Research Center for Allergy, Immunology, and Microbiome (A.I.M.), China Medical University Children’s Hospital, Taichung 404333, Taiwan; 6 Graduate Institute of Educational Administration and Policy, National Chengchi University, Taipei City 116011, Taiwan

**Keywords:** Mediterranean diet, type 2 diabetes mellitus, HbA1c, fasting plasma glucose, BMI, meta-analysis, randomized controlled trials

## Abstract

**Background:** Type 2 diabetes mellitus (T2DM) is a growing global health challenge requiring effective dietary management strategies. While the Mediterranean diet shows promise for cardiovascular and metabolic health, the last comprehensive meta-analysis of randomized controlled trials (RCTs) examining its effects on glycemic control and body mass index (BMI) in T2DM was published in 2015. Multiple RCTs, including culturally adapted interventions with extended follow-up, have since been completed, but remain unsynthesized. **Methods:** We conducted a systematic review and meta-analysis following PRISMA 2020 guidelines (PROSPERO: CRD420251147035), searching PubMed, Web of Science, and Embase from inception through 17 August 2025. Unlike previous syntheses that combined observational cohorts or mixed dietary approaches, our analysis focused strictly on RCTs in adults with established T2DM and incorporated trials published after 2015. We included RCTs comparing Mediterranean diet interventions against non-Mediterranean control diets in adults with T2DM. Primary outcomes included glycated hemoglobin (HbA1c), fasting plasma glucose (FPG), and body mass index (BMI). Secondary outcomes comprised low-density lipoprotein cholesterol (LDL-C), systolic blood pressure (SBP), and diastolic blood pressure (DBP). Pooled effects were estimated using random-effects models. **Results:** Eleven RCTs (10 publications) involving diverse populations met inclusion criteria. Compared with control diets, Mediterranean diet interventions showed reductions in HbA1c (mean difference [MD] −0.307%, 95% CI: −0.451 to −0.163), FPG (MD −0.845 mmol/L, 95% CI: −1.307 to −0.384), and BMI (MD −0.828 kg/m^2^, 95% CI: −1.4 to −0.256). Secondary analyses revealed reductions in LDL-C (MD −8.060 mg/dL, 95% CI: −14.213 to −1.907), SBP (MD −5.130 mmHg, 95% CI: −10.877 to 0.617), and DBP (MD −2.008 mmHg, 95% CI: −3.027 to −0.989). Sensitivity analyses supported stability of findings, with no substantial publication bias detected. Subgroup analyses revealed geographic variation in blood pressure responses, with greater benefits observed in non-Mediterranean populations. **Conclusions:** Mediterranean dietary patterns were associated with modest improvements in glycemic control, body composition, and cardiometabolic risk factors among adults with T2DM. The cultural adaptability of this approach may support implementation in clinical practice, though larger multicenter trials with standardized protocols and extended follow-up remain necessary.

## 1. Introduction

Type 2 diabetes mellitus (T2DM) prevalence continues to rise globally, driven largely by increasing obesity rates and sedentary behavior [1]. Although pharmacological advances have expanded treatment options, dietary modification remains fundamental for achieving optimal glycemic control and preventing long-term complications. The challenge lies in identifying sustainable dietary patterns that patients can maintain while still achieving clinically meaningful metabolic improvements.

The Mediterranean dietary pattern has received considerable research attention. Characterized by abundant plant-based foods, such as vegetables, fruits, legumes, and whole grains with olive oil as the primary fat source, moderate consumption of fish and dairy, and limited red meat intake, this pattern differs markedly from typical Western diets. Observational studies and several intervention trials have linked Mediterranean dietary patterns to improved cardiovascular outcomes and metabolic health [2], although the specific magnitude of benefit for glycemic parameters and weight management in individuals with established T2DM remains incompletely characterized [3,4].

The most recent comprehensive meta-analysis examining Mediterranean diet effects on glycemic outcomes and body mass index (BMI) in T2DM was published by Huo et al. [5], incorporating nine trials available through February 2014 and reporting modest, but statistically significant improvements in HbA1c (−0.47%), fasting glucose (−0.55 mmol/L), and BMI (−0.29 kg/m^2^). However, more than a decade has passed since that synthesis, and multiple additional RCTs—including culturally adapted Mediterranean-style interventions and trials with follow-up durations of up to 8 years—have since been completed. These newer trials were not included in earlier evidence syntheses. Subsequent meta-analyses have either focused on observational cohort studies [6,7], examined limited subsets of Mediterranean diet trials [8], or addressed diabetes prevention rather than management in individuals with established disease [9]. Importantly, no recent meta-analysis has exclusively synthesized RCT evidence in adults with established T2DM, while concurrently evaluating glycemic control, body composition, lipid profiles, and blood pressure outcomes, nor have potential effect moderators such as geographic region been systematically explored.

Therefore, the objective of this study was to perform a systematic review and meta-analysis of RCTs, from database inception through 17 August 2025, to quantify the effect of Mediterranean dietary patterns on glycated hemoglobin (HbA1c), fasting plasma glucose (FPG), BMI, and cardiometabolic outcomes in adults with T2DM, as well as to explore potential moderators such as geographic region and intervention type. Unlike previous syntheses that combined observational cohorts or mixed dietary approaches, our analysis focuses strictly on RCTs in adults with established T2DM and incorporates trials published after 2015. This approach allowed us to (1) increase precision of effect size estimates through inclusion of recent high-quality trials, (2) concurrently evaluate weight, glycemic, lipid, and blood pressure outcomes within a unified analytical framework, and (3) explore moderators of heterogeneity such as geographic region and intervention type—analyses not comprehensively reported in earlier RCT-focused meta-analyses. By synthesizing the most current RCT evidence with rigorous methodology, we aimed to provide more precise effect estimates to better inform clinical nutrition recommendations for T2DM management.

## 2. Methods

This systematic review and meta-analysis was conducted in accordance with PRISMA 2020 guidelines (Preferred Reporting Items for Systematic Reviews and Meta-Analyses) [10], and the protocol was prospectively registered in PROSPERO https://www.crd.york.ac.uk/prospero/ (accessed on 13 September 2025) with Registration Number: CRD420251147035.

### 2.1. Search Strategy

Literature searches were conducted in PubMed, Embase, and Web of Science from database inception to 17 August 2025, restricted English-language publications. No date limits were applied. Predefined search strategies combined controlled vocabulary and free-text terms related to the Mediterranean diet, type 2 diabetes, and RCTs. Full search strings for each database are provided in Supplementary File S1. Additionally, we searched ClinicalTrials.gov (accessed on 17 August 2025) to identify unpublished trials or supplementary data for potentially eligible RCTs. Importantly, we also constructed a sensitive search strategy focusing on the population (type 2 diabetes mellitus) and intervention (Mediterranean or Mediterranean-style diet). In line with Cochrane guidance and to avoid unnecessary loss of sensitivity, we did not include explicit comparator or outcome terms in the database search; instead, comparator diets and predefined outcomes were applied at the full-text screening and eligibility stages. Similarly, we did not apply an RCT filter at the search level; randomized design was required during study selection.

### 2.2. Eligibility Criteria

We included RCTs enrolling adults (≥18 years) with a clinical diagnosis of T2DM. Eligible interventions focused on Mediterranean or Mediterranean-style dietary patterns, operationally defined as high consumption of fruits, vegetables, legumes, whole grains, nuts, and olive oil; moderate intake of fish and dairy; and low consumption of red or processed meat. Multi-component lifestyle interventions were included if Mediterranean dietary counseling constituted a primary component.

Control groups received non-Mediterranean dietary interventions such as low-fat diets, standard diabetic diets, or usual care, with a minimum intervention duration of 12 weeks. Studies were required to report at least one predefined outcome. Primary outcomes included HbA1c, FPG, and BMI; secondary outcomes included LDL-C, systolic blood pressure (SBP), and diastolic blood pressure (DBP).

We excluded non-randomized studies, single-arm trials, interventions combining diet with pharmacologic or surgical treatments, reports lacking sufficient data for effect size estimation, and interventions without clearly defined Mediterranean dietary components. When multiple publications reported overlapping data from the same RCT, we retained the most complete or most recent report to avoid double-counting.

### 2.3. Data Extraction and Risk of Bias Assessment

Two reviewers independently extracted data using standardized electronic forms, and disagreements were resolved through discussion with a third reviewer. Extracted variables included publication year, study location, sample size, participant characteristics, intervention and control diet descriptions, follow-up duration, and quantitative outcome data. When clarification or additional data were required, corresponding authors were contacted.

Risk of bias was evaluated using the Revised Cochrane Risk-of-Bias tool for RCTs (RoB 2) [11] https://methods.cochrane.org/bias/resources/rob-2-revised-cochrane-risk-bias-tool-randomized-trials (accessed on 17 August 2025), assessing five domains: (1) randomization process, (2) deviations from intended interventions, (3) missing outcome data, (4) outcome measurement, and (5) selection of reported results. Each domain was rated as low risk, some concerns, or high risk, and an overall risk-of-bias judgment was assigned for each study.

### 2.4. Study Selection

**Figure 1:**
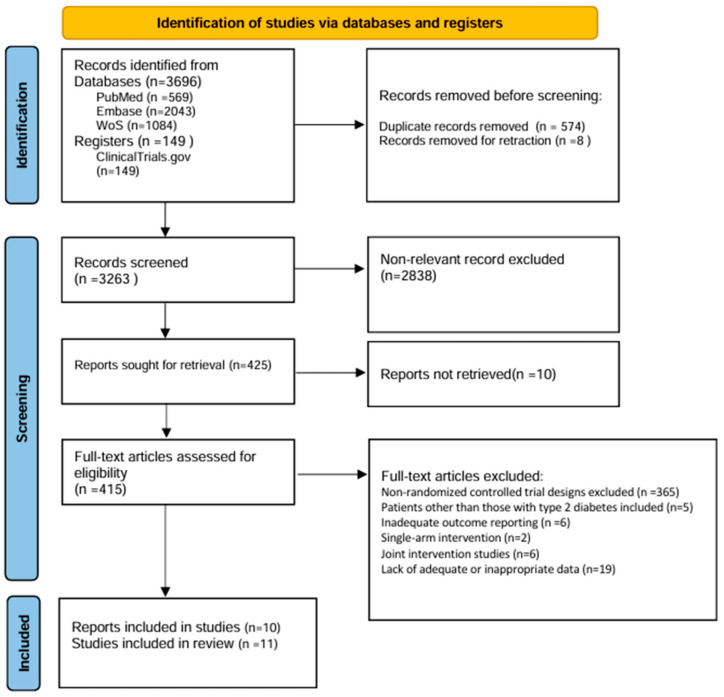
Flow diagram of literature search and study selection.

Figure 1 presents the PRISMA 2020 flow diagram summarizing study identification, screening, eligibility assessment, and inclusion. Database searches identified 3696 records (PubMed *n* = 569; Embase *n* = 2043; Web of Science *n* = 1084), supplemented by 149 records from trial registries. After removing duplicates (*n* = 574) and retracted articles (*n* = 8), 3263 records were screened. Screening of title and abstract excluded 2838 records, leaving 425 for full-text assessment; however, 10 full-text reports could not be retrieved.

Among the 415 full-text articles assessed for eligibility, 404 were excluded for the following reasons: 365 non-randomized designs, 5 studies enrolling non-T2DM populations, 2 duplicate publications from the same trial, 6 with inadequate outcome data, 2 single-arm interventions, 6 multi-component interventions where a Mediterranean diet was not the primary component, and 19 with missing or inappropriate data. Finally, only 10 publications reporting on 11 RCTs met all inclusion criteria (Figure 1).

### 2.5. Statistical Analysis

Statistical analyses were performed using Comprehensive Meta-Analysis software (version 4.0; Biostat, Englewood, NJ, USA). For continuous outcomes (HbA1c [%], FPG [mmol/L], BMI [kg/m^2^], LDL-C [mg/dL], SBP [mmHg], DBP [mmHg]), pooled mean differences and 95% confidence intervals (CIs) were calculated using random-effects models [12].

When studies reported standard error (SE), confidence intervals (CI), or interquartile ranges (IQR) instead of standard deviation (SD), we converted these statistics to SD using standard formulas from the Cochrane Handbook (version 6.5) https://www.cochrane.org/authors/handbooks-and-manuals/handbook (accessed on 1 September 2025) [13]. When SDs were not available, they were derived from other summary statistics (e.g., CI width). These procedures ensured all eligible studies contributed to pooled estimates even when variance measures were not directly reported.

Heterogeneity was assessed using Cochran’s Q test and quantified with the I^2^ statistic [14], with thresholds of 25%, 50%, and 75% representing low, moderate, and high heterogeneity, respectively. Sensitivity and subgroup analysis were performed to explore potential sources of high heterogeneity. Publication bias was evaluated through visual funnel plot inspection and Egger’s regression test [15], with the trim-and-fill method applied to estimate the potential impact of missing studies when asymmetry was detected. Statistical significance was set at two-tailed *p* < 0.05.

Included trials were conducted in Europe, Asia, and North America, with sample sizes ranging from 11 to 280 participants and intervention durations from 3 months to 8 years. All participants had a confirmed diagnosis of T2DM. Most interventions emphasized Mediterranean dietary patterns, sometimes combined with additional lifestyle counseling, whereas control groups received low-fat diets, standard diabetic diets, or usual care. Key characteristics of the included trials are summarized in Table 1.

## 3. Results

### 3.1. Risk of Bias Assessment

Assessment of risk of bias using the RoB 2 assessment tool indicated that 4 of the trials (Toobert [24]; Maiorino [21]; Esposito [20]; Alonso-Domínguez [16]) had a low overall risk of bias when assessed against all RoB 2 domains. Trials assessed as having some concerns for bias primarily occurred because of deviation from the intended intervention due to participant and personnel un-blinding, which is an inherent issue in diet-based intervention studies. None of the trials were deemed to be at high overall risk of bias (Supplemental Figures S1 and S2). We have modified the description to include the objective limitation that although the randomization process was generally satisfactory, details on allocation concealment and blinding were often not reported.

### 3.2. Primary Outcomes

#### 3.2.1. Glycated Hemoglobin (HbA1c)

**Figure 2:**
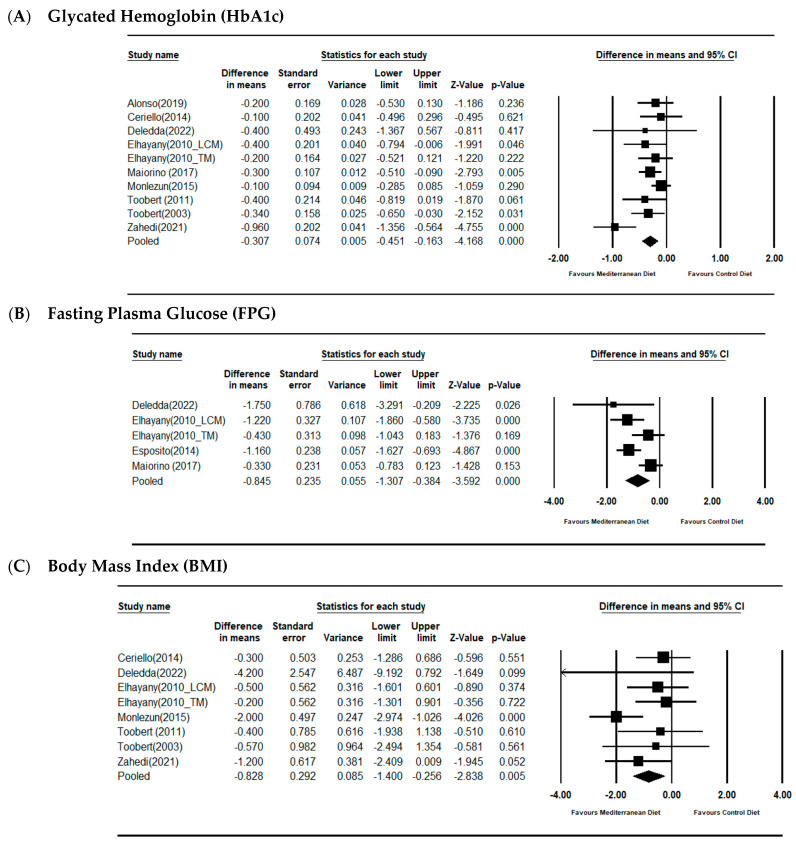
Forest plots assessing the effects of MSD on HbA1c (**A**), FPG (**B**) and BMI (**C**); expressed as the mean differences between the intervention and the control. The area of each square is proportional to the inverse of the variance of the MD. Horizontal lines represent 95% CIs. Diamonds represent pooled estimates from random-effects analysis [16,17,18,19,20,21,22,23,24,25].

Pooled analysis using random-effects models showed a statistically significant reductio n in HbA1c with Mediterranean diet interventions compared with controls, with a mean difference of −0.307% (95% CI: −0.451 to −0.163; *p* < 0.001) and low-to-moderate heterogeneity (I^2^ = 47.4%) (Figure 2A). Funnel plot inspection and Egger’s test indicated no substantial publication bias (see Supplementary Figure S3a–f).

#### 3.2.2. Fasting Plasma Glucose (FPG)

Mediterranean dietary interventions were associated with significantly lower FPG levels in random-effects analysis, with pooled mean difference of −0.845 mmol/L (95% CI: −1.307 to −0.384; *p* < 0.001) and moderate heterogeneity (I^2^ = 63.37%) (Figure 2B).

#### 3.2.3. Body Mass Index (BMI)

Anthropometric outcomes showed modest but statistically significant BMI reduction favoring Mediterranean diet interventions (MD −0.828 kg/m^2^; 95% CI: −1.4 to −0.256; *p* = 0.005) using random-effects models, with low heterogeneity (I^2^ = 34.81%) (Figure 2C).

### 3.3. Secondary Outcomes

#### 3.3.1. Low-Density Lipoprotein Cholesterol (LDL-C)

**Figure 3:**
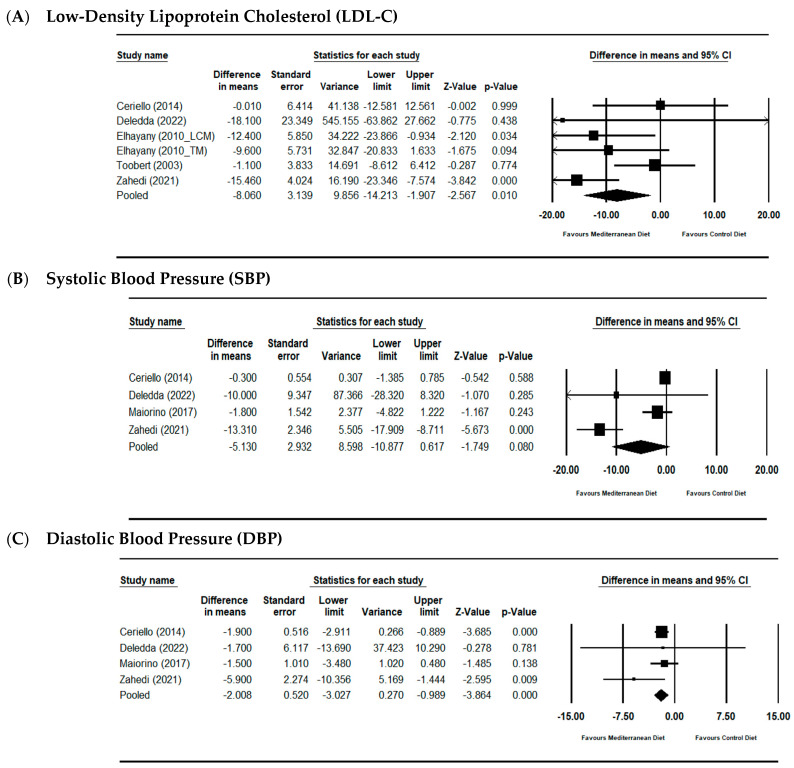
Forest plots assessing the effects of MSD on LDL-C (**A**), SBP (**B**) and DBP (**C**); expressed as the mean differences between the intervention and the control. The area of each square is proportional to the inverse of the variance of the MD. Horizontal lines represent 95% CIs. Diamonds represent pooled estimates from random-effects analysis [17,18,19,21,23,25].

Mediterranean dietary patterns showed favorable LDL-C effects compared with control diets (MD −8.060 mg/dL; 95% CI: −14.213 to −1.907; *p* = 0.01) using random-effects models, with low-to-moderate heterogeneity (I^2^ = 44.8%) (Figure 3A).

#### 3.3.2. Systolic Blood Pressure (SBP)

Pooled analysis demonstrated lower SBP with Mediterranean diet interventions (MD −5.130 mmHg; 95% CI: −10.877 to 0.617; *p* = 0.08), though considerable heterogeneity was observed (I^2^ = 69.459%) (Figure 3B).

#### 3.3.3. Diastolic Blood Pressure (DBP)

For DBP, Mediterranean dietary interventions showed small but statistically significant reductions compared to control diets (MD −2.008 mmHg; 95% CI: −3.027 to −0.989; *p* < 0.001) with no heterogeneity detected (I^2^ = 6.96%) (Figure 3C).

### 3.4. Publication Bias

We found no evidence of substantial publication bias from Egger’s test (*p* > 0.05) for any outcome examined (Supplementary Table S1).

### 3.5. Sensitivity and Subgroup Analyses

Leave-one-out sensitivity analyses demonstrated that no single trial disproportionately influenced the overall significance or direction of the pooled effects for HbA1c, FPG, BMI, LDL-C, SBP, or DBP. Excluding any individual study did not materially alter the magnitude or statistical significance of the summary estimates for either primary or secondary outcomes. Across all iterations, pooled effects consistently favored Mediterranean diet interventions, supporting the robustness and stability of the findings.

Heterogeneity of participant outcomes existed in terms of fasting plasma glucose (FPG) and systolic blood pressure (SBP), so subgroup analyses were conducted to investigate potential moderators. Subgroup analyses explored geographic region and intervention context.

For FPG, the pooled effect across five studies was significant (estimated point = −0.787 mmol/L, *p* < 0.001), and each subgroup (Israel and Italy) showed individually significant effects. Importantly, the test for heterogeneity between subgroups was not significant (*p* for subgroup difference > 0.05), indicating that geographical region (Mediterranean vs. non-Mediterranean) did not moderate the effect on fasting glucose. These findings suggest that improvements in glycemic control are broadly reproducible across different geographic and cultural contexts.

A pooled analysis for SBP demonstrated some degree of decreased blood pressure; however, there was also substantial variation among individual studies. An exploratory sub-group analysis using geographic region as the basis for the subgroups found that these variations were statistically significant (*p* < 0.001). There appeared to be two distinct patterns of change across the sub-groups; trials located outside of the Mediterranean showed substantial and statistically significant decreases in SBP while trials located within the Mediterranean area showed little or no change in SBP. This pattern may indicate that population-based dietary habits, culture, etc., can function as effect modifiers and provide greater benefit when individuals transition from western-style diets. These results must be viewed cautiously due to the low number of trials used in the analysis and additional research would be required to test and support this hypothesis.

## 4. Discussion

This meta-analysis of trial data spanning the last decade provided recent and accurate estimates of how the Mediterranean Diet impacts the management of T2DM through the use of a large body of evidence. Our study found that the Mediterranean Diet had significantly positive effects on HbA1c, FPG and BMI and also had beneficial effects for LDL-C and BP and as such demonstrated wide-ranging cardiometabolic health benefits. We also performed leave-one-out sensitivity analyses to support the reliability of our results.

### 4.1. Previous Evidence Comparison

Our findings extend the seminal work of Huo et al. [5], which analyzed nine trials available through February 2014 and reported HbA1c reduction of −0.47%, FPG reduction of −0.55 mmol/L, and BMI reduction of −0.29 kg/m^2^. By including four additional RCTs and incorporating over 10 years of new evidence, we provide updated estimates with improved precision. Unlike previous syntheses combining observational and interventional studies [6,7] or examining mixed dietary patterns [8], our analysis focuses strictly on RCTs in adults with established T2DM, minimizing confounding and strengthening causal inference. The HbA1c reduction we observed (0.30%) aligns closely with Huo et al. [5]’s findings, reinforcing consistency across time periods while offering greater precision due to increased sample size.

Recent work by Zheng et al. [26] reported similar glycemic benefits, though their analysis included fewer trials and did not comprehensively evaluate cardiometabolic outcomes. Our meta-analysis uniquely combines assessment of glycemic parameters, anthropometric measures, and cardiovascular risk factors within a single framework, enabling integrated evaluation of Mediterranean diet’s multifaceted metabolic effects. Furthermore, our systematic subgroup analyses explored moderators of heterogeneity such as geographic region and intervention type—analyses not comprehensively reported in earlier RCT-focused meta-analyses. These explorations revealed important effect modifiers: while improvements in glycemic control appeared broadly reproducible across geographic contexts, blood pressure responses differed significantly by baseline dietary culture. Our subgroup analysis indicates that populations with baseline diets higher in processed foods, sodium, and saturated fats may experience greater cardiometabolic benefit when transitioning to a Mediterranean-style pattern, whereas populations already consuming Mediterranean-like diets may show attenuated blood pressure responses.

### 4.2. Mechanistic Considerations and Clinical Relevance

The included randomized controlled trials (RCT) did not have an intent to study the mechanisms of biology for the observed improvement in metabolism; however, it is possible that the metabolic changes could be due to a variety of synergistic mechanisms. The high monounsaturated fat content from olive oil and nuts combined with the abundance of polyphenols was demonstrated by previous mechanistic studies to improve endothelial function and reduce oxidative stress. In addition, higher fiber consumption has been proposed to improve insulin sensitivity through modulation of the gut microbiota and the production of short chain fatty acids while also providing a sensation of fullness. Finally, as many components of the Mediterranean dietary pattern have an anti-inflammatory profile, this too could add to the potential to regulate metabolism and would explain why lower levels of inflammatory markers were noted in several of the included trials.

The clinical impact of a drop in HbA1c of about 0.3% is relatively small; nonetheless, epidemiologic evidence from the United Kingdom Prospective Diabetes Study (UKPDS) supports that even such small drops can be associated with a statistically significant lower rate of development of microvascular complications if they are sustained over time [27].

In conjunction with the observed positive changes in lipid profile and blood pressure levels associated with Mediterranean diets, it is reasonable to conclude that Mediterranean diets have an impact on reducing overall cardiovascular risk, as cardiovascular disease continues to be the number one cause of death in individuals diagnosed with Type 2 diabetes.

### 4.3. Implementation Challenges

Despite demonstrated efficacy, several implementation barriers warrant consideration. Most included trials provided intensive dietary counseling or structured programs not readily available in routine clinical practice. Socioeconomic factors affect access to key Mediterranean diet components, particularly extra-virgin olive oil, fresh fish, and abundant produce. Cost analyses from several trials suggested Mediterranean dietary patterns may be more expensive than typical diets, potentially limiting accessibility [28]. Baseline dietary quality varied considerably across trials; individuals consuming poor-quality Western diets at baseline may derive greater benefits than those already following relatively healthful patterns, as suggested by our subgroup findings.

Adaptation of cultural practices is associated with both opportunity and challenge. The data collected from the subgroup analysis provide evidence that the core Mediterranean dietary guidelines can be applied in different cultures if they take into account local cuisine and available food. Additional research is required to support the development of Mediterranean diets adapted for a particular culture or region while ensuring adherence to key components of the Mediterranean diet.

### 4.4. Strengths and Limitations

Strengths of this meta-analysis include strict inclusion of RCTs only, incorporation of recent trials published after 2015, concurrent evaluation of multiple cardiometabolic outcomes within a unified analytical framework, comprehensive sensitivity analyses demonstrating robustness of findings, and systematic exploration of effect modifiers including geographic region and intervention context.

Limitations warrant consideration. First, geographic concentration represents a significant constraint. Most included trials were conducted in Mediterranean or Western countries; relatively few trials enrolled participants from East Asian or other non-Mediterranean dietary cultures. This may limit generalizability to global populations. Specifically, the FPG analysis was limited to Israel and Italy, both Mediterranean countries, and the lack of data from non-Mediterranean regions (e.g., Asia, Africa, Latin America) restricts ability to fully delineate the role of global geographical variables in glycemic control. Geographic heterogeneity emerged as a critical driver of pooled results, particularly for systolic blood pressure, where stark differences were observed between Mediterranean and non-Mediterranean subgroups.

Second, heterogeneity in intervention intensity may have influenced findings. Interventions ranged from simple dietary education to comprehensive lifestyle programs, whereas control conditions varied (low-fat diet, ADA-standard diet, routine care). Differences in behavioral support intensity may partially explain heterogeneity, particularly for systolic blood pressure. This variability in intervention and control designs, coupled with variations in dietary components and comparison diets, may have influenced overall findings.

Third, follow-up duration varied substantially across trials. Several trials were short-term (≤6 months), while others reported multi-year follow-up (up to 8 years). Longer-term cardiometabolic durability, particularly for blood pressure and lipid outcomes, requires further confirmation in pragmatic, real-world settings. The variation in intervention duration may have affected observed effect sizes.

Fourth, reporting of secondary outcomes was incomplete. Some RCTs did not provide data for all cardiometabolic endpoints (e.g., LDL-C, SBP, DBP), which introduces imprecision in pooled estimates and constrained the ability to conduct subgroup analyses for these outcomes. This incomplete reporting contributed to wider confidence intervals for some secondary endpoints.

Fifth, small sample sizes in several trials (range: 11 to 280 participants) may have limited statistical power for detecting smaller effects or conducting comprehensive subgroup analyses. Sixth, definitions of Mediterranean diet varied across trials, though all adhered to core principles of high plant-based food consumption, olive oil as primary fat source, and limited red meat intake.

Future multi-site studies with standardized dietary protocols, extended follow-up periods, larger sample sizes, and representation from diverse geographic regions—particularly non-Mediterranean populations—are necessary to establish durability of findings and improve generalizability across diverse cultures and geographical settings. Additionally, pragmatic trials evaluating implementation strategies in real-world clinical settings would help address the translation gap between efficacy and effectiveness, and clarify optimal approaches for delivering Mediterranean diet interventions with varying levels of behavioral support intensity.

Finally, our literature search was restricted to English-language publications, which may introduce a potential language bias.

## 5. Conclusions

In summary, this PRISMA 2020-guided meta-analysis of 11 randomized controlled trials provides updated evidence that Mediterranean dietary patterns are associated with clinically meaningful improvements in glycemic control, body composition, and cardiometabolic risk factors in adults with type 2 diabetes mellitus. Compared with control diets, Mediterranean interventions reduced HbA1c by 0.307%, fasting plasma glucose by 0.845 mmol/L, and BMI by 0.828 kg/m^2^, with additional benefits for LDL cholesterol and blood pressure. These modest reductions translate to meaningful long-term reductions in microvascular and cardiovascular complication risk.

Importantly, subgroup analyses revealed that blood pressure responses differ by baseline dietary culture, with greater benefits observed in populations consuming highly processed, high-sodium diets. This suggests Mediterranean dietary patterns may be particularly valuable for patients transitioning from typical Western diets, supporting its role as a culturally adaptable, evidence-based nutritional strategy in diabetes management.

However, implementation challenges related to cost, accessibility, and intensive dietary support require consideration. Larger pragmatic trials with extended follow-up and diverse populations—particularly from non-Mediterranean regions—are warranted to establish optimal implementation strategies and confirm long-term durability of benefits across diverse cultural and geographical settings.

## Figures and Tables

**Table 1:** Characteristics of included studies.

Study ID (First Author, Year)	Country	Participants	Sample Size (T/C)	Age (Years, T/C; Sex)	Duration, Design	Mediterranean Diet Intervention	Control Diet	Outcomes
Alonso-Domínguez [16]	Spain	Patients with Type 2 Diabetes Mellitus (T2DM)	T: 102/C: 102	Mean: 60.6 ± 8.1 (Overall); 45.6% Female (Overall)	12 months, controlled and randomized trial	Multifactorial Intervention (Focused on Mediterranean Diet Adherence)	Brief advice about healthy eating and physical activity	HbA1c, BMI, SBP, DBP, LDL-C
Ceriello [17]	Spain	Type 2 diabetic patients	T: 12/C: 12	Not reported (Total: 17M/7F)	3 months, randomized trial	Mediterranean Diet using olive oil	Low-fat diet	HbA1c, BMI, SBP, DBP, LDL-C
Deledda [18]	Italy	Drug-Naïve T2DM patients with Overweight/Obesity	T: 5 (MEDI)/C: 6 (KETO)	T: 45–65 (Range); 3M/2F/C: 45–65 (Range); 3M/3F	3 months, randomized	Hypocaloric Mediterranean Diet (MD)	Very Low-Calorie Ketogenic Diet (VLCKD)	HbA1c, FPG, BMI, LDL-C, SBP, DBP
Elhayany [19] (LCM)	Israel	Overweight T2DM patients	T: 85 (LCM)/C: 85 (ADA)	T: 55.5 ± 6.5/C: 56.0 ± 6.1; Sex T: 31M/30F/C: 27M/28F	1 year, randomized intervention	Low-Carbohydrate Mediterranean (LCM) diet	American Diabetes Association (ADA) diet	HbA1c, FPG, BMI, SBP, DBP, LDL-C
Elhayany [19] (TM)	Israel	Overweight T2DM patients	T: 89 (TM)/C: 85 (ADA)	T: 57.4 ± 6.1/C: 56.0 ± 6.1; Sex T: 35M/28F/C: 27M/28F	8.1 years, randomized controlled trial (follow-up)	Traditional Mediterranean (TM) diet	ADA diet	HbA1c, FPG, BMI, SBP, DBP, LDL-C
Esposito [20]	Italy	Overweight, middle-aged men and women with newly diagnosed T2D	T: 108/C: 107	Mean: 52.2 years (Overall); 50% Female (T)/51.5% Female (C)	8.1 years, parallel, two-arm, single-center trial	Low-Carbohydrate Mediterranean Diet (LCMD)	Low-fat diet	HbA1c, SBP, DBP, LDL-C, BMI
Maiorino [21]	Italy	Men and women with newly diagnosed T2D	T: 108/C: 107	Mean: 52 years (Overall); 50% Female (T)/51.5% Female (C)	6 months, pilot randomized controlled trial	Low-Carbohydrate Mediterranean Diet	Low-fat diet	HbA1c, SBP, LDL-C, BMI
Monlezun [22]	USA	Patients with Type 2 Diabetes (T2D)	T: 18/C: 9	Mean: 62 years (Overall); 67% Female (Overall)	6 months, randomized clinical trial	Teaching Kitchen (Culinary Medicine: Mediterranean Diet-based curriculum)	Registered Dietitian (RD)-led Medical Nutrition Therapy (MNT)	HbA1c, DBP, Total Cholesterol (LDL not specifically separated)
Toobert [23]	USA	Postmenopausal women with Type 2 Diabetes (T2DM)	T: 163/C: 116	Mean age not reported; All Postmenopausal Female	24 months, randomized controlled trial	Mediterranean Lifestyle Program (MLP: Low-saturated fat Mediterranean diet + others)	Usual care	HbA1c, BMI, SBP, DBP, LDL-C
Toobert [24]	USA	Latinas with Type 2 Diabetes (T2DM)	T: 142/C: 138	T: 55.6 ± 9.7/C: 58.7 ± 10.3; All Female	6 months, randomized controlled trial	Culturally adapted Mediterranean Diet Lifestyle Program	Enhanced usual diabetes care	HbA1c, BMI
Zahedi [25]	Iran	Well-controlled Type 2 Diabetes Mellitus patients	T: 105/C: 123	T: 49.4 ± 11.3/C: 46.8 ± 11.2; Sex T: 18F/4M/C: 18F/3M	6 months, randomized controlled trial	Mediterranean educational intervention (8 sessions) + classic Mediterranean diet	Routine diabetes care	FPG, HbA1c, BMI, SBP, DBP, LDL-C

Note: T = treatment group; C = control group; HbA1c = glycated hemoglobin; BMI = body mass index; SBP = systolic blood pressure; DBP = diastolic blood pressure; LDL-C = low-density lipoprotein cholesterol; M = male; F = female; FPG = fasting plasma glucose; LDL = low-density lipoprotein.

## Data Availability

Data supporting the findings of this study are available from the corresponding author upon reasonable request.

## Supplementary Materials

The following supporting information can be downloaded at https://www.mdpi.com/article/10.3390/nu17243908/s1, File S1: Full search strategy. Table S1. Evaluation of heterogeneity and publication bias for studies include in the meta analysis. Figure S1. Risk of bias per domain across included randomized controlled trail. Figure S2. Risk of bias per domain and per randomized controlled trail. Figure S3. Funnel plots for the different outcomes.
