# Supplementary material for: Impact of the Mediterranean Diet on Glycemic Control, Body Mass Index, Lipid Profile, and Blood Pressure in Type 2 Diabetes: A Meta-Analysis of Randomized Controlled Trials
Source: Nutrients. 2025 Dec 13;17(24):3908. doi: 10.3390/nu17243908 (PMC12735701; doi:10.3390/nu17243908)
Supplement: Supplementary file 1 [file nutrients-17-03908-s001.zip › nutrients-3937980-supplementary.pdf]

Supplementary File S1: Full search strategy

PubMed

#1

((("Diet, Mediterranean"[MeSH Terms]) OR ("Mediterranean-style diet"[Title/Abstract])) OR ("Mediterranean-type diet"[Title/Abstract])) OR ("Mediterranean dietary pattern"[Title/Abstract])

#2

((("Diabetes Mellitus, Type 2"[MeSH Terms]) OR ("Type 2 diabetes"[Title/Abstract])) OR (T2D[Title/Abstract])) OR ("Non-insulin-dependent diabetes"[Title/Abstract])

#3 #1 AND #2

Embase

#1

'mediterranean diet'/exp OR (mediterranean-style diet*:ti,ab) OR (mediterranean-type diet*:ti,ab) OR (mediterranean dietary pattern:ti,ab)

#2

'type 2 diabetes mellitus'/exp OR ('type 2 diabetes':ti,ab OR t2d:ti,ab OR 'non-insulin-dependent diabetes':ti,ab)

#3 #1 AND #2

WoS

#1

TS=(("Mediterranean diet") OR ("Mediterranean-style diet") OR ("Mediterranean-type diet") OR ("Mediterranean dietary pattern"))

#2

TS=(("Type 2 diabetes mellitus") OR ("type 2 diabetes") OR (T2D) OR ("non-insulin-dependent diabetes"))

#3 #1 AND #2

Supplementary Table S1:

Evaluation of heterogeneity and publication bias for studies include in the meta analysis

	P value of Cochrane *Q* test	I^2 statistic	*P* value of the Egger's test
Primary outcomes			
HbA_{1c} (%)	0.047	47.419%	0.199
FPG (mmol/L)	0.027	63.371%	0.481
BMI (kg/m^2)	0.150	34.812%	0.629
Second outcomes			
LDL cholesterol (mmol/L)	0.107	44.816%	0.835
Systolic blood pressure (mmHg)	0.000	90.092%	0.280
Diastolic blood pressure (mmHg)	0.358	6.96%	0.514

Supplementary Figures S1 Risk of bias per domain across included randomized controlled trail.

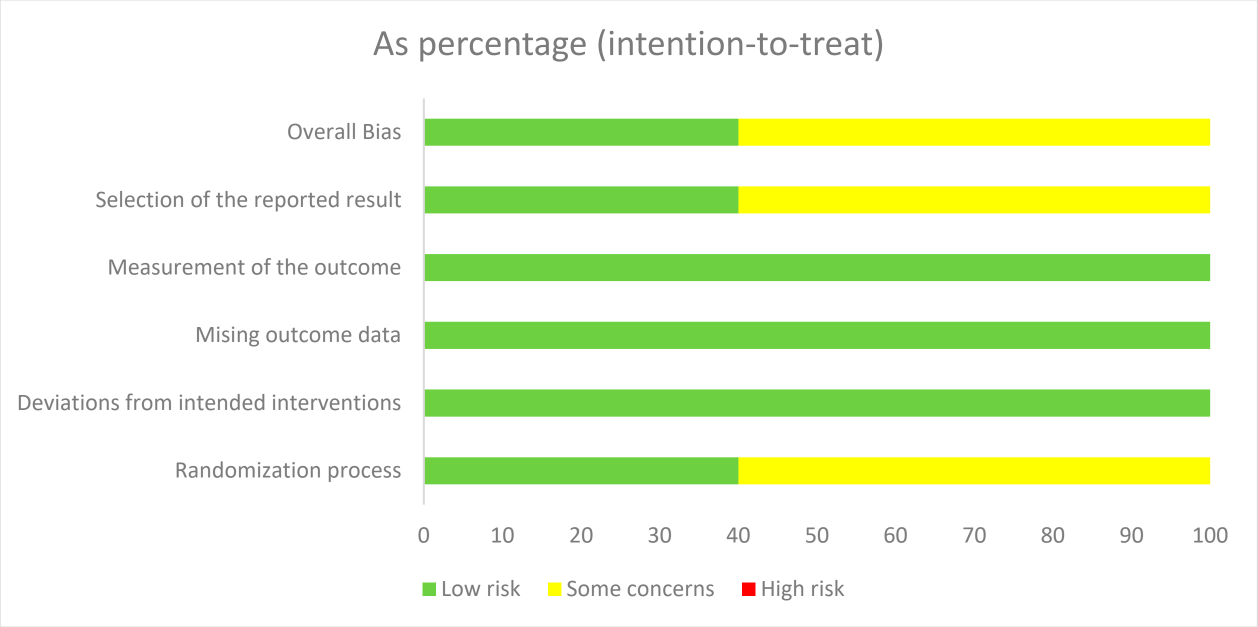

Supplemental Figures S2 Risk of bias per domain and per randomized controlled trail.

Study ID	Experimental	Comparator	Outcome	Weight	D1	D2	D3	D4	D5	Overall
Alonso (2019)	Food workshop+Smartphone app+Waks+advice	Standardized advice only	Change in MEDAS(primary)/DQI(Secindary)	1	+	+	+	+	+	+
Ceriello (2014)	MedFDiet+Olive oil(12 weeks)	Low fat diet(12 weeks)	FMD,oxidative stress,inflammation,antioxidant capacity, GLP-1 effect	1	!	+	+	+	!	!
Deledda (2022)	ketogenic diet	Mediterranean diet	Gut microbiota composition	1	!	+	+	+	!	!
Elhayany (2010)	Low-carbohydrate Mediterranean diet	Traditional Mediterranean diet, ADA diet	HbA1C, BMI	1	!	+	+	+	!	!
Espocito (2014)	Low-carbohydrate Mediterranean diet	Low-fat diet	Time to initiation of DM drug therapy	1	+	+	+	+	+	+
Maiorino (2017)	Mediterranean diet	Low-fat diet	EPCs,CIMT,HbA1C	1	+	+	+	+	+	+
Monlezun (2015)	GCCM	MNT	HbA1C	1	!	+	+	+	!	!
Toobert (2003)	Mediterranean lifestyle program	Usual use	HbA1C,BMI...	1	!	+	+	+	!	!
Toobert (2011)	culturally adapted Mediterranean lifestyle program	Usual diabetes care	Psychosocial,HbA1C,BMI	1	+	+	+	+	+	+
Zahedi (2021)	Mediterranean diet educational intervention group	Routine diabetes care	FBS, HbA1C	1	!	+	+	+	!	!

\+ Low risk
! Some concerns
\- High risk

D1 Randomisation process
D2 Deviations from the intended interventions
D3 Missing outcome data
D4 Measurement of the outcome
D5 Selection of the reported result

Supplemental Figures S3 Funnel plots for the different outcomes.

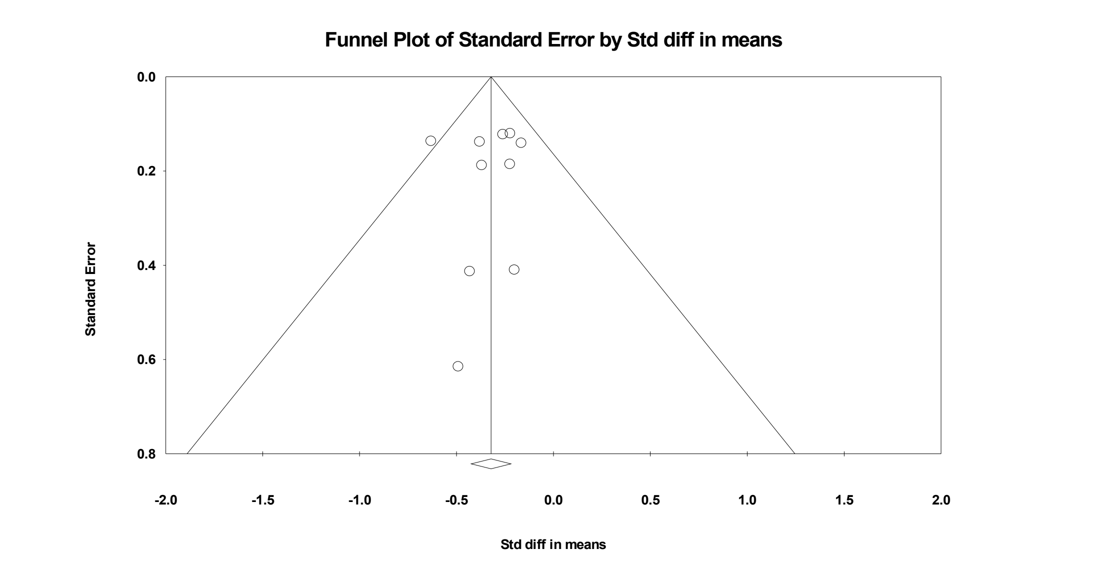

HbA1C

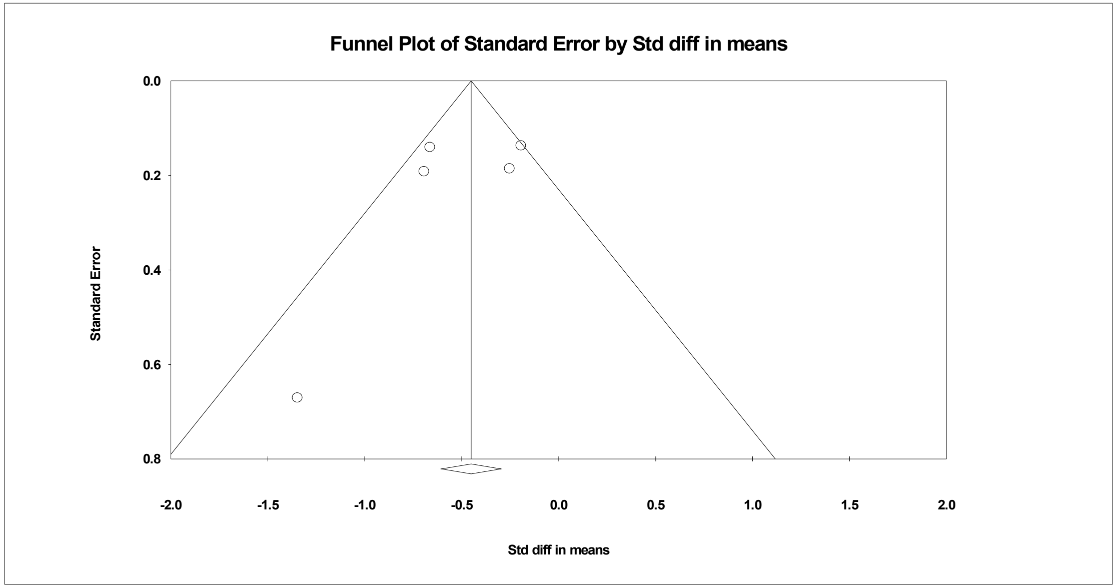
Funnel Plot of Standard Error by Std diff in means
Standard Error
0.0
0.2
0.4
0.6
0.8
-2.0
-1.5
-1.0
-0.5
0.0
0.5
1.0
1.5
2.0
Std diff in means

FPG

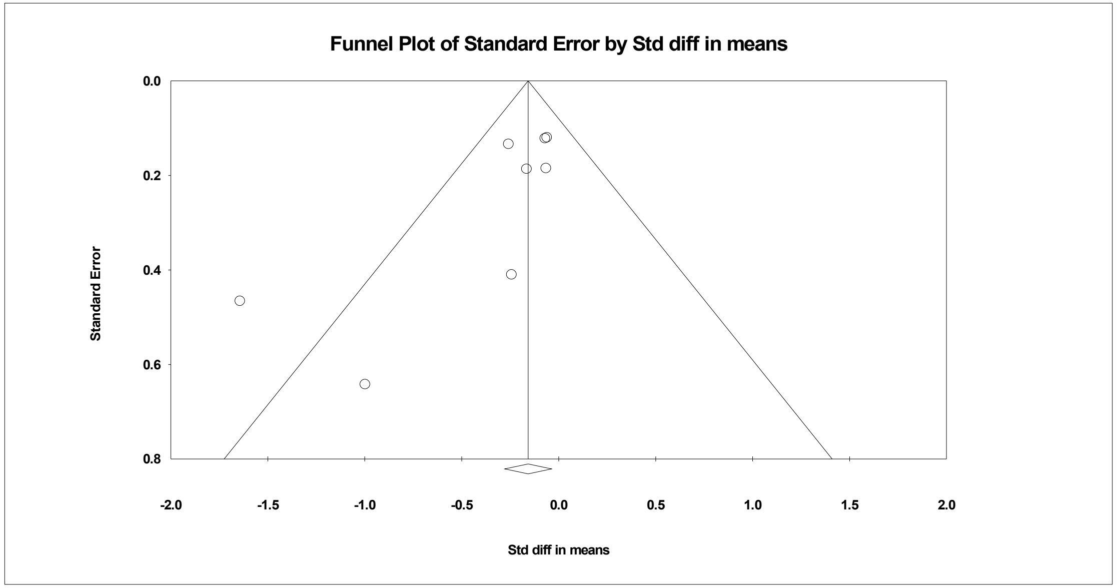
Funnel Plot of Standard Error by Std diff in means
Standard Error
0.0
0.2
0.4
0.6
0.8
-2.0
-1.5
-1.0
-0.5
0.0
0.5
1.0
1.5
2.0
Std diff in means

BMI

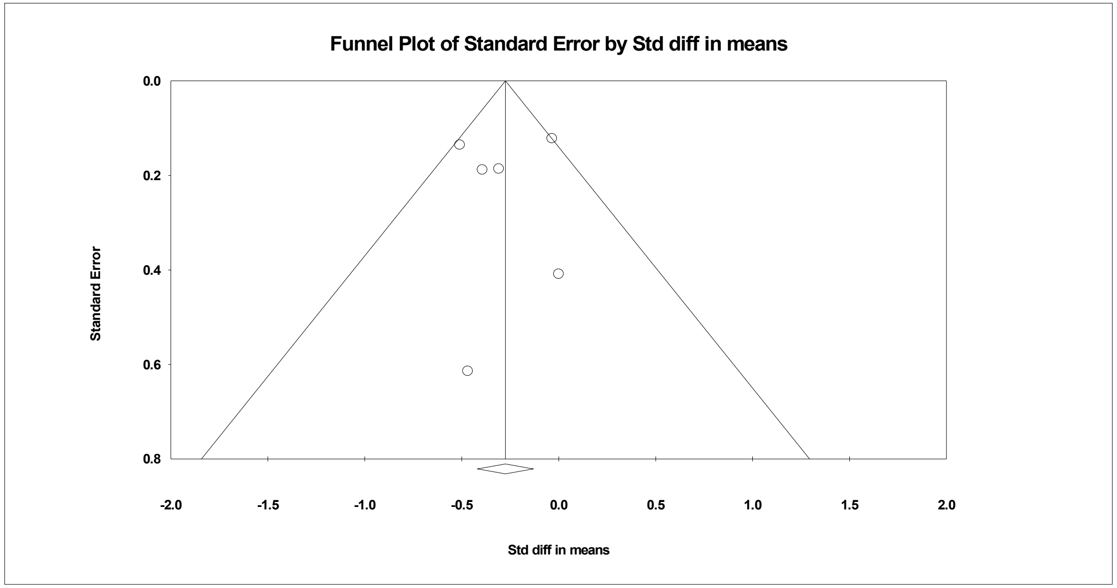
Funnel Plot of Standard Error by Std diff in means
Standard Error
0.0
0.2
0.4
0.6
0.8
-2.0
-1.5
-1.0
-0.5
0.0
0.5
1.0
1.5
2.0
Std diff in means

LDL-C

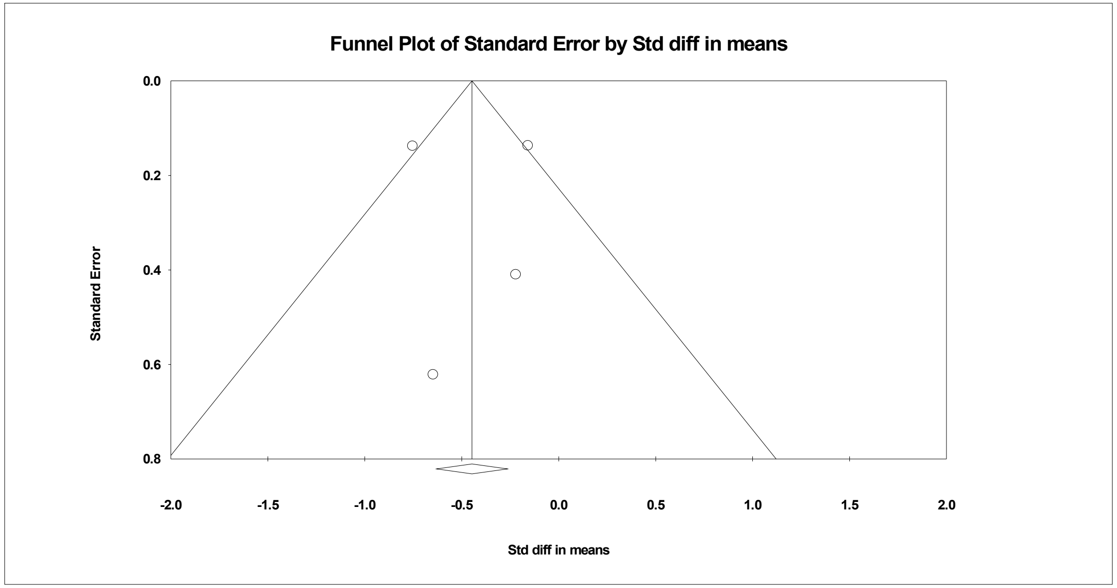
Funnel Plot of Standard Error by Std diff in means
Standard Error
0.0
0.2
0.4
0.6
0.8
-2.0
-1.5
-1.0
-0.5
0.0
0.5
1.0
1.5
2.0
Std diff in means

SBP

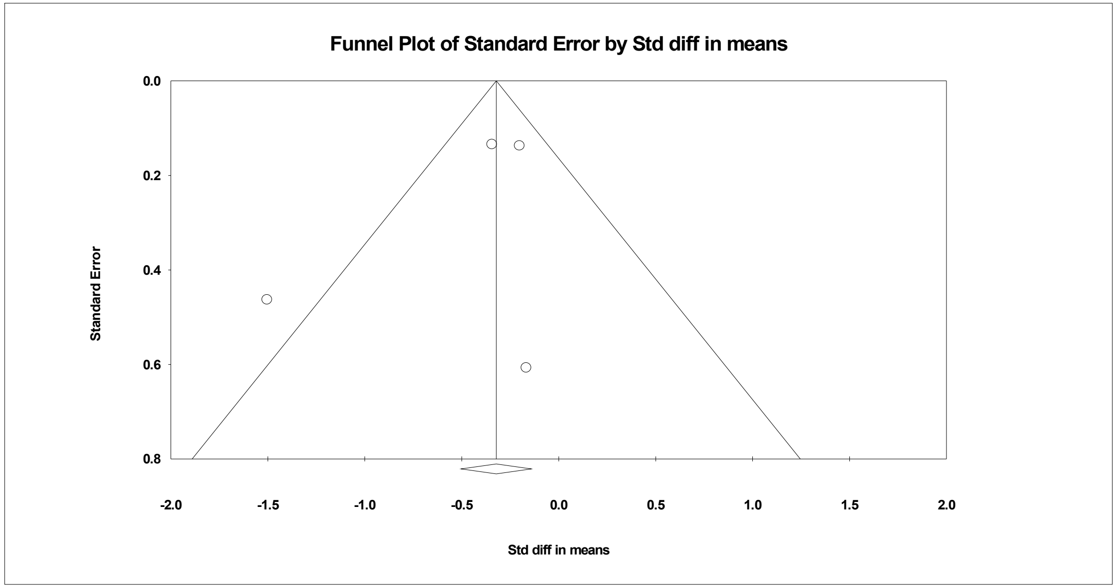
Funnel Plot of Standard Error by Std diff in means
Standard Error
0.0
0.2
0.4
0.6
0.8
-2.0
-1.5
-1.0
-0.5
0.0
0.5
1.0
1.5
2.0
Std diff in means

DBP
